# In vitro activity of dolutegravir against wild-type and integrase inhibitor-resistant HIV-2

**DOI:** 10.1186/s12977-015-0146-8

**Published:** 2015-02-05

**Authors:** Robert A Smith, Dana N Raugi, Charlotte Pan, Papa Salif Sow, Moussa Seydi, James I Mullins, Geoffrey S Gottlieb, the University of Washington-Dakar HIV-2 Study Group

**Affiliations:** Center for Emerging and Re-emerging Infectious Diseases (CERID) and Department of Medicine, Allergy & Infectious Diseases, University of Washington, Seattle, WA USA; Service des Maladies Infectieuses, CHNU de Fann, Dakar, Senegal; Departments of Microbiology, Medicine and Laboratory Medicine, University of Washington, Seattle, WA USA; Department of Global Health, University of Washington, Seattle, WA USA

## Abstract

**Background:**

Dolutegravir recently became the third integrase strand transfer inhibitor (INSTI) approved for use in HIV-1–infected individuals. In contrast to the extensive dataset for HIV-1, in vitro studies and clinical reports of dolutegravir for HIV-2 are limited. To evaluate the potential role of dolutegravir in HIV-2 treatment, we compared the susceptibilities of wild-type and INSTI-resistant HIV-1 and HIV-2 strains to the drug using single-cycle assays, spreading infections of immortalized T cells, and site-directed mutagenesis.

**Findings:**

HIV-2 group A, HIV-2 group B, and HIV-1 isolates from INSTI-naïve individuals were comparably sensitive to dolutegravir in the single-cycle assay (mean EC_50_ values = 1.9, 2.6, and 1.3 nM, respectively). Integrase substitutions E92Q, Y143C, E92Q + Y143C, and Q148R conferred relatively low levels of resistance to dolutegravir in HIV-2_ROD9_ (2- to 6-fold), but Q148K, E92Q + N155H, T97A + N155H and G140S + Q148R resulted in moderate resistance (10- to 46-fold), and the combination of T97A + Y143C in HIV-2_ROD9_ conferred high-level resistance (>5000-fold). In contrast, HIV-1_NL4-3_ mutants E92Q + N155H, G140S + Q148R, and T97A + Y143C showed 2-fold, 4-fold, and no increase in EC_50_, respectively, relative to the parental strain. The resistance phenotypes for E92Q + N155H, and G140S + Q148R HIV-2_ROD9_ were also confirmed in spreading infections of CEM-ss cells.

**Conclusions:**

Our data support the use of dolutegravir in INSTI-naïve HIV-2 patients but suggest that, relative to HIV-1, a broader array of replacements in HIV-2 integrase may enable cross-resistance between dolutegravir and other INSTI. Clinical studies are needed to evaluate the efficacy of dolutegravir in HIV-2–infected individuals, including patients previously treated with raltegravir or elvitegravir.

## Findings

Human immunodeficiency virus type 2 (HIV-2) infection is a significant public health problem in West Africa and has been reported in other countries with socioeconomic ties to the region [1]. Dual HIV-1/HIV-2 infection also occurs in areas where the viruses co-circulate [2-6]. Historically, clinical outcomes of antiretroviral therapy in HIV-2 and HIV-1/HIV-2 dually positive patients have been poor, with high rates of immuno-virologic failure and emergent multidrug resistance [7-11]. Newer classes of antiretrovirals (ARV) with anti–HIV-2 activity could represent substantial improvements to the current therapeutic picture [12,13].

A growing body of evidence suggests that integrase strand transfer inhibitors (INSTI) might be particularly useful for HIV-2 treatment. Raltegravir and elvitegravir are both potent inhibitors of HIV-2 replication in culture [14-18], and case reports and small case series (primarily involving ARV-experienced individuals) indicate that raltegravir and elvitegravir can reduce HIV-2 viral loads when combined with other suppressive ARV [19-32]. As with HIV-1, changes at integrase residues Y143, Q148 or N155, together with other secondary replacements in the integrase protein (*i.e.*, E92Q, T97A, G140S, and possibly others), confer resistance to raltegravir in HIV-2 [26,28-35]. The emergence of resistance to elvitegravir has not yet been reported in HIV-2-infected individuals but will likely involve these same three pathways based on studies of HIV-1 [36-50] and the extensive cross-resistance seen between raltegravir and elvitegravir in HIV-2 in culture [15,17]. Clinical trials of raltegravir- and elvitegravir-containing regimens for first-line HIV-2 treatment are now underway and are expected to yield data within the next few years (NCT01605890, NCT02150993, NCT02180438).

A third strand transfer inhibitor, dolutegravir, was recently approved by the United States Food and Drug Administration (FDA) for use in both INSTI-naïve and INSTI-experienced HIV-1 patients. Although dolutegravir has been extensively evaluated for HIV-1 treatment, few studies have examined its potential use in HIV-2–infected individuals. Charpentier and colleagues reported that HIV-2_ROD,_ HIV-1_BRU_, and eight HIV-2 isolates from INSTI-naïve patients were comparably susceptible to dolutegravir in spreading infections of peripheral blood mononuclear cells (PBMC) (EC_50_ = 0.2–4 nM) and that three HIV-2 isolates from raltegravir-treated individuals with consensus integrase genotypes G140S + Q148R (group A), G140T + Q148R + N155H (group A), and T97A + Y143C (group H) were 63-, 9-, and 5-fold resistant to dolutegravir, respectively, in PBMC [51]. In addition, the manufacturer of dolutegravir (ViiV Healthcare) reported that EC_50_ values against three clinical isolates of HIV-2 ranged from 0.09 nM to 0.61 nM in PBMC assays, and that combinations of substitutions A153G + N155H + S163G and E92Q + T97A + N155H + S163D in HIV-2 integrase conferred 4-fold decreases in dolutegravir susceptibility, while E92Q + N155H and G140S + Q148R resulted in 8.5-fold and 17-fold decreases, respectively [52].

The ability of dolutegravir to inhibit strains resistant to other INSTI is of particular importance–in HIV-1, mutations Q148H/K/R, together with secondary changes in the integrase protein, confer resistance to dolutegravir in cell culture [38,47,53-55], and other mutations associated with diminished in vitro susceptibility to dolutegravir have been reported [56-61]. In contrast, dolutegravir is fully active against HIV-1 variants bearing Y143 or N155 mutations (with or without secondary changes) in both single-cycle and spreading infection assays [38,47,53-55], although it should be noted that Y143 and N155 mutants have been observed in raltegravir-experienced patients who subsequently failed dolutegravir-based regimens [62,63]. In the VIKING-3 trial, dolutegravir response rates (<50 HIV-1 RNA copies/ml at week 24) declined from 79% (n = 100/126) for patients without Q148 mutations at baseline (including those with N155H, Y143C/H/R, T66A, E92Q, or historical evidence of INSTI resistance), to 58% (21/36) for patients with Q148 plus one additional secondary mutation, to 24% (5/21) for those with Q148 plus two or more secondary mutations [64]. Importantly, drug resistance testing is not widely available in West Africa, and thus, dolutegravir usage in many HIV-2–infected patients, including INSTI-experienced individuals, will depend on an algorithmic approach to treatment. To date, there are only two reports of dolutegravir treatment for HIV-2 infection ([65,66]; n = 2 and 13 patients, respectively), with limited duration of follow-up.

In the present study, we examined the activity of dolutegravir against wild-type and INSTI-resistant HIV-2 strains using an indicator cell assay that restricts viral replication to a single cycle [15]. This methodology enables a direct comparison of HIV-1 and HIV-2 drug susceptibility while avoiding potential confounders such as differences in replication rates, infectivity, cytopathic potential and cell-to-cell spread.

**Figure 1:**
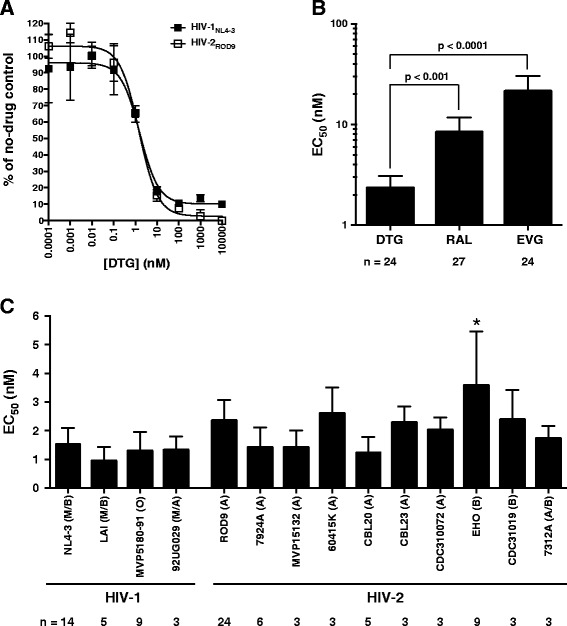
**Susceptibility of wild-type HIV-1 and HIV-2 isolates to dolutegravir in the single-cycle assay. (A)** Representative dose-response profiles for HIV-1_NL4-3_ and HIV-2_ROD9_. Virus stocks were generated by transient transfection of chloroquine-treated 293T/17 cultures with plasmids pNL4-3 and pROD9, respectively. Dolutegravir was obtained from Selleck Chemicals, Inc. Titers are expressed as the percentage of no-drug (solvent-only) controls and are the means of two independent cultures at each drug concentration. Curve fits were generated using the sigmoid dose-response function of Prism version 6.0 (GraphPad Software, Inc.). **(B)** Comparison of the activity of dolutegravir (DTG), raltegravir (RAL), and elvitegravir (EVG) against wild-type HIV-2_ROD9_. Values for RAL and EVG include data from two previously-published studies of HIV-2 from our group [14,15] plus additional determinations; all data were obtained using the single-cycle assay. Bars indicate mean 50% effective concentrations (EC_50_); the number of independent determinations (n) for each strain is shown below the x-axis. P values were obtained via analysis of variance (ANOVA) of log_10_-transformed EC_50_ values with Tukey’s post test (Prism v6.0). No cytotoxic effects were observed in dolutegravir-treated MAGIC-5A cultures at concentrations as high as 10,000 nM. **(C)** Activity of dolutegravir against wild-type HIV-1 and HIV-2 isolates. Group/subtype designations are shown in parentheses. HIV-1_NL4-3_ and HIV-2_ROD9_ were generated as in panel A. HIV-2_EHO_ was kindly provided by Jan McClure (University of Washington). The remaining isolates were obtained from the National Institutes of Health AIDS Reagent Program (www.aidsreagent.org). *, significantly greater than HIV-2_ROD9_, HIV-2_MVP15132_, HIV-2_CBL20_, and all HIV-1 isolates listed (p < 0.05, ANOVA with Tukey’s post test). In all panels, error bars indicate standard deviations.

We initially compared the dolutegravir sensitivities of viruses derived from two prototypic full-length molecular clones: pNL4-3 (HIV-1 group M, subtype B) and pROD9 (HIV-2 group A). In head-to-head single-cycle assays, these two strains showed nearly identical dose-response profiles (Figure 1A). Over multiple assays runs, the mean EC_50_ values for dolutegravir (± standard deviation) were 1.5 ± 0.6 nM for HIV-1_NL4-3_ and 2.3 ± 0.7 nM for HIV-2_ROD9_ (n = 14 and 24 determinations, respectively). Dolutegravir was 3.6-fold more potent than raltegravir and 9.1-fold more potent than elvitegravir against HIV-2_ROD9_ (Figure 1B). Other isolates from ARV-naïve individuals displayed levels of dolutegravir sensitivity comparable to HIV-1_NL4-3_ and HIV-2_ROD9_ (Figure 1C). The aggregate EC_50_ values for HIV-1, HIV-2 group A, and HIV-2 group B were 1.3 ± 0.2 nM, 1.9 ± 0.5 nM, and 2.6 ± 0.9 nM, respectively. When subjected to a one-way ANOVA, only the comparison between HIV-1 and HIV-2 group B reached statistical significance (p < 0.05); this modest difference was attributable to the slightly higher EC_50_ for HIV-2_EHO_ (3.6 ± 1.9 nM) (Figure 1C). Notably, HIV-2_EHO_ integrase contains a glutamate at position 146, whereas other HIV-2 isolates (as well as HIV-1) encode glutamine at this site [67,68]. Substitutions at Q146 have been observed in HIV-1 following in vitro selections with elvitegravir and other, investigational INSTI [18,69,70]. To our knowledge, Q146 mutations have not been observed in HIV-2 variants selected in culture, nor have they been reported in HIV-2 patients treated with INSTI-based regimens.

**Figure 2:**
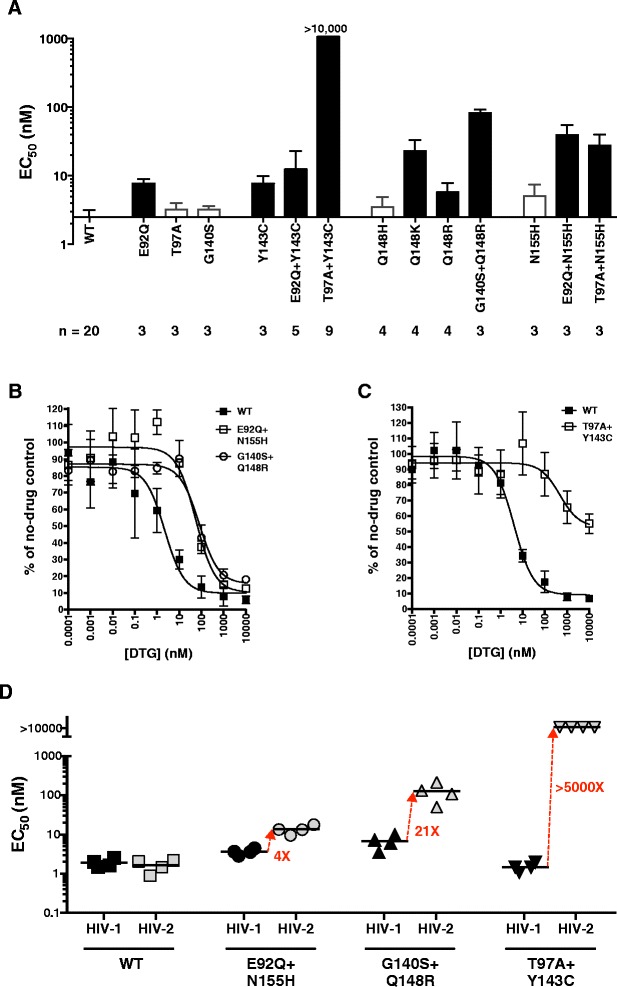
**Antiviral activity of dolutegravir against site-directed mutants of HIV-1 and HIV-2 integrase in the single-cycle assay. (A)** EC_50_ values for wild-type (WT) HIV-2_ROD9_ and HIV-2_ROD9_ integrase mutants generated in the full-length pROD9 molecular clone. Shaded bars indicate strains that are significantly different from wild-type (p < 0.05, ANOVA of log_10_-transformed EC_50_ values with Tukey’s post test; Prism v6.0). The number of independent determinations (n) for each strain is shown below the x-axis. **(B)** and **(C)** Representative dose-response profiles for WT, E92Q + N155H, G140S + Q148R and T97A + Y143C HIV-2_ROD9_. Data are the means of three independent cultures at each dose of dolutegravir (DTG). Curve fits were generated as described in Figure 1A. **(D)** Comparative analysis of equivalent site-directed mutants of HIV-1 and HIV-2 integrase. Each point is the result of a single dose-response assay performed as described in Figure 1A. Horizontal bars indicate the mean EC_50_ values for each strain. Red arrows and text indicate fold increases in the mean EC_50_ values for HIV-2 relative to HIV-1. ANOVA results for these data are described in the main text. In all panels, error bars represent standard deviations.

To examine potential resistance pathways in HIV-2, we tested the activity of dolutegravir against a panel of site-directed mutants of HIV-2_ROD9_ using the single-cycle assay. These variants contained amino acid replacements in the integrase protein that are associated with raltegravir and elvitegravir treatment; their phenotypes with respect to raltegravir and elvitegravir susceptibility have been reported elsewhere [14,15]. Single amino acid changes T97A, G140S, Q148H and N155H had no significant effect on dolutegravir sensitivity (p > 0.05, ANOVA; Figure 2A). In contrast, mutants E92Q, Y143C, E92Q + Y143C, Q148K, and Q148R were resistant to dolutegravir, with EC_50_ values 2.3–9.3-fold greater than that of the parental strain (Figure 2A), and variants E92Q + N155H, T97A + N155H and G140S + Q148R exhibited 11–33-fold resistance to the drug (p < 0.05, ANOVA; Figure 2A and B). In experiments with T97A + Y143C HIV-2_ROD9_, dolutegravir concentrations as high as 10 μM failed to reduce viral replication by 50% (Figure 2A and C; EC_50_ > 10 μM), although modest dose-dependent inhibition was apparent at doses ≥100 nM (Figure 2C). Altogether, nine of the 13 HIV-2 integrase mutants tested were resistant to dolutegravir in the single-cycle assay (Figure 2A).

We also evaluated the dolutegravir sensitivities of E92Q + N155H, T97A + Y143C, and G140S + Q148R HIV-2_ROD9_ in three-day spreading infections of immortalized T cells (CEM-ss). These assays were preformed as previously described for the MT-2 T cell line [14]. The resultant EC_50_ values for the parental strain, E92Q + N115H, and G140S + Q148R were 0.24, 2.1 and 73 nM, respectively, indicating 8.8-fold resistance to dolutegravir for E92Q + N115H and 300-fold resistance for G140S + Q148R. Despite repeated attempts using high multiplicities of infection (≥0.1) and prolonged incubation times (up to seven days), CEM-ss cultures inoculated with T97A + Y143C HIV-2_ROD9_ failed to produce detectable levels of infectious virus, indicating a severe fitness defect. This result is consistent with the poor replication capacity previously reported for T97A + Y143C HIV-2_ROD9_ [15].

Lastly, we performed a head-to-head comparison of the phenotypes conferred by E92Q + N155H, G140S + Q148R, and T97A + Y143C in HIV-1_NL4-3_ and HIV-2_ROD9_ in the single-cycle assay. G140S + Q148R resulted in slight resistance to dolutegravir in HIV-1_NL4-3_ (3.5-fold; p <0.01, ANOVA), whereas E92Q + N155H and T97A + Y143C had no statistically significant effect in the HIV-1_NL4-3_ background (Figure 2D). These data are entirely consistent with previous studies of HIV-1 [38,47,53,54]. In contrast, HIV-2_ROD9_ mutants E92Q + N155H, G140S + Q148R, and T97A + Y143C were all resistant to dolutegravir (p < 0.0001, ANOVA) and showed EC_50_ values 4-, 21- and >5000-fold greater than those seen for equivalent mutants of HIV-1_NL4-3_, respectively (Figure 2D). EC_50_ and fold change values for all HIV-1_NL4-3_ and HIV-2_ROD9_ integrase mutants tested in this study, together with the corresponding EC_50_ values for the parental wild-type clones, are compiled in Table 1.

**Table 1:** **Compilation of EC**_**50**_ **and fold change values for site-directed mutants of HIV-2**_**ROD9**_ **and HIV-1**_**NL4-3**_ **integrase**

**HIV Type**	**Strain**	**EC**_**50**_ **for DTG (nM)** ^**a**^	**n** ^**b**^	**Fold Change** ^**c**^
HIV-2	Wild-type	2.3 ± 0.7	24	
	E92Q	**7.7 ± 1.2**	3	3
	T97A	3.2 ± 0.8	3	1
	G140S	3.2 ± 0.8	3	1
	Y143C	**7.7 ± 2.2**	3	3
	Q148H	3.5 ± 1.4	4	1
	Q148K	**23 ± 10**	4	10
	Q148R	**5.7 ± 2.1**	4	2
	N155H	5.0 ± 2.4	3	2
	E92Q + Y143C	**15 ± 10**	5	6
	T97A + Y143C	**>10000**	13	>5000
	G140S + Q148R	**108 ± 54**	7	46
	E92Q + N155H	**25 ± 17**	7	10
	T97A + N155H	**27 ± 13**	3	12
HIV-1	Wild-type	1.5 ± 0.6	14	
	T97A + Y143C	1.5 ± 0.4	4	1
	G140S + Q148R	**6.8 ± 2.7**	4	4
	E92Q + N155H	**3.6 ± 0.7**	4	2

^a^50% effective concentration of dolutegravir (DTG) as measured in the MAGIC-5A single-cycle assay. Values were compiled from the data used to generate Figures 2A and 2D and are expressed as means ± standard deviations. Numbers shown in bold type are significantly greater than the values for the corresponding wild-type strains (p < 0.05; ANOVA of log_10_-transformed EC_50_ values with Tukey’s post-test; performed in Prism version 6.0, GraphPad Software, Inc.).

^b^Number of independent determinations for each strain.

^c^Fold change in EC_50_ relative to the corresponding wild-type strain.

Taken together, our results indicate that prototypic HIV-1 and HIV-2 strains, as well as HIV-1 and HIV-2 isolates from INSTI-naïve individuals, are comparably sensitive to dolutegravir in a single cycle of viral replication in MAGIC-5A indicator cells (Figure 1). These findings complement previous data from spreading infections of PBMC [51]–using a different methodology and target cell type–and suggest that dolutegravir would be an appropriate treatment choice for INSTI-naïve HIV-2 patients when combined with other HIV-2–active ARV. We also report the effects of raltegravir-associated mutations on dolutegravir susceptibility using site-directed mutagenesis of genetically-defined HIV-1 and HIV-2 molecular clones (pNL4-3 and pROD9, respectively). Our analysis shows that equivalent amino acid changes in the integrase proteins of HIV-1 and HIV-2 can have differing effects on dolutegravir susceptibility (Figure 2D) and that, in HIV-2_ROD9_, integrase changes Q148K, T97A + Y143C, E92Q + N155H, T97A + N155H, and G140S + Q148R confer moderate to high levels of dolutegravir resistance (≥10-fold; Figure 2A–C and Table 1). We cannot exclude the possibility that the resistance levels observed in our site-directed HIV-2 mutants are specific to the ROD9 molecular clone, as the genetic context within integrase can have a substantial impact on the phenotypic expression of INSTI resistance [71,72]. For example, in the aforementioned study by Charpentier et al. [51], a group H HIV-2 isolate with T97A + Y143C was only 5-fold resistant to dolutegravir (this isolate differs from HIV-2_ROD9_ at 24 of 293 amino acid sites in the integrase protein). In addition, the roles of novel INSTI-associated changes (i.e, H51Y, G118R, F121Y, E138A/K, and R263K; [26,34,57-61,63,73]) remain to be determined in HIV-2, and the level of dolutegravir resistance in vitro that correlates with virologic failure in HIV-2–infected patients is unknown. Nonetheless, our findings suggest that, relative to HIV-1, a broader array of amino acid changes in HIV-2 integrase might facilitate cross-resistance between dolutegravir and other INSTI. Phenotypic drug resistance testing of HIV-2 isolates from raltegravir- and elvitegravir-treated patients should be performed as these drugs become more widely available in West Africa, and studies of dolutegravir-based regimens should be conducted in HIV-2–infected individuals, including patients previously treated with other INSTI.

## Abbreviations

HIV-1: Human immunodeficiency virus type 1
HIV-2: Human immunodeficiency virus type 2
ARV: antiretroviral
INSTI: Integrase strand transfer inhibitor
EC_50_: 50% effective concentration
DTG: Dolutegravir
RAL: Raltegravir
EVG: Elvitegravir
FDA: Food and drug administration
ANOVA: Analysis of variance
